# Familial Mediterranean Fever Intertwined With a Diagnosis of Crohn’s Disease: A Case Report

**DOI:** 10.7759/cureus.78380

**Published:** 2025-02-02

**Authors:** Catarina Ferreira, Eduardo Macedo, Ana S Ramoa Oliveira, Ana Rita Marques

**Affiliations:** 1 Department of Oncology, Hospital de Braga, Braga, PRT; 2 Department of Internal Medicine, Hospital de Braga, Braga, PRT

**Keywords:** anakinra, autoinflammatory diseases, colchicine, crohn’s disease, familial mediterranean fever, infliximab

## Abstract

Autoinflammatory diseases are a group of disorders caused by the dysregulation of the innate immune system, leading to episodes of systemic inflammation, with familial Mediterranean fever (FMF) being the most common among them. Other inflammatory disorders have recently been associated with FMF, including inflammatory bowel disease, specifically Crohn’s disease (CD). While the gastrointestinal involvement in FMF is not the most typical presentation, both FMF and CD can share clinical features that challenge their diagnosis and treatment. We present a case of a man in his 50s who initially presented with a fever and was subsequently diagnosed with FMF, followed by appropriate treatment. However, he received a later diagnosis of CD after experiencing therapeutic failure and the development of severe gastrointestinal symptoms. Significant clinical improvement occurred only after initiating targeted therapy for CD with infliximab. This case illustrates the complexity and the diagnostic and therapeutic challenges that arise when these two diseases coexist, emphasizing the importance of a multidisciplinary approach to achieve better clinical outcomes.

## Introduction

Autoinflammatory diseases (AIDs) are a growing group of disorders caused by the dysregulation of the innate immune system, leading to episodes of systemic inflammation [1]. Familial Mediterranean fever (FMF) is the oldest and most frequent AID. Predominantly diagnosed in populations originating from the Mediterranean basin, it is an autosomal recessive hereditary periodic fever syndrome characterized by self-limited episodes of fever and systemic inflammation commonly associated with pleural and abdominal serositis and arthritis. The MEFV gene was first described in 1997 as the gene responsible for FMF, and it encodes mutant protein pyrin, an important player in the innate immune system and a component of inflammasome that leads to an exaggerated inflammatory response through uncontrolled interleukin-1 (IL-1) production [2-5]. FMF is mainly a clinical diagnosis based on the Tel-Hashomer criteria, but it can be supported by genetic testing. The treatment is based on colchicine or IL-1 inhibitors, like anakinra [4,5].

Other inflammatory and autoimmune disorders have been recently associated with FMF and MEFV gene mutations, probably due to mutual dysregulations in the immune system, such as inflammatory bowel disease (IBD), ankylosing spondylitis, Behçet’s disease, multiple sclerosis, and rheumatoid arthritis [3,5]. IBD is a chronic inflammatory disease of the intestinal tract that includes ulcerative colitis and Crohn’s disease (CD). Although the gastrointestinal involvement in FMF has been considered quite atypical, both FMF and IBD can share clinical features, like recurrent episodes of fever, abdominal pain, and arthralgia, so the concurrence of these two diseases can be a challenge to diagnose and treat [6]. The coexistence of FMF and CD is rare, with very few previous reports of adult patients with both FMF and CD.

We present a case of a man in his 50s who experienced a three-month history of fever, asthenia, and night sweats. He was initially diagnosed and treated for FMF, but two years later, he received a diagnosis of CD following therapeutic failure and the development of severe gastrointestinal symptoms.

## Case presentation

We describe a 53-year-old man with multiple cardiovascular risk factors (essential hypertension, type 2 diabetes mellitus, and class I obesity), hepatic steatosis, and a familiar history of CD (mother and three siblings), who presented to the emergency department complaining of asthenia, night sweats, and fever during the past three months (temperature rise to 38°C, one to two days of duration, and one to two episodes per week). No anorexia, weight loss, arthralgia, abdominal pain, diarrhea, or respiratory symptoms were reported. Physical examination was unremarkable. Bloodwork revealed no anemia or leukocytosis but increased inflammatory parameters (Table 1).

**Table 1:** Laboratory results and evolution over time (+): increased; (-): decreased

Lab parameters (units)	Patient values				Reference range
	Admission	12 months after starting colchicine	Six months after starting anakinra + colchicine association	Six months after anakinra’s suspension	
Hemoglobin (g/dL)	13.5	14.5 (+)	14.0 (-)	12.5 (-)	13.5-17.0
White blood cells (/uL)	8,200	5,800 (-)	6,500 (+)	11,500 (+)	4,500-11,000
C-reactive protein (mg/L)	43	12 (-)	16.50 (+)	141 (+)	<5.0
Erythrocyte sedimentation rate (mm/hour)	52	7 (-)	17 (+)	77 (+)	<15
Ferritin (ng/mL)	1,240	726 (-)	669 (-)	1,179 (+)	30-322
Fecal calprotectin (ug/g)	-	-	-	>1,500	<50

**Figure 1:**
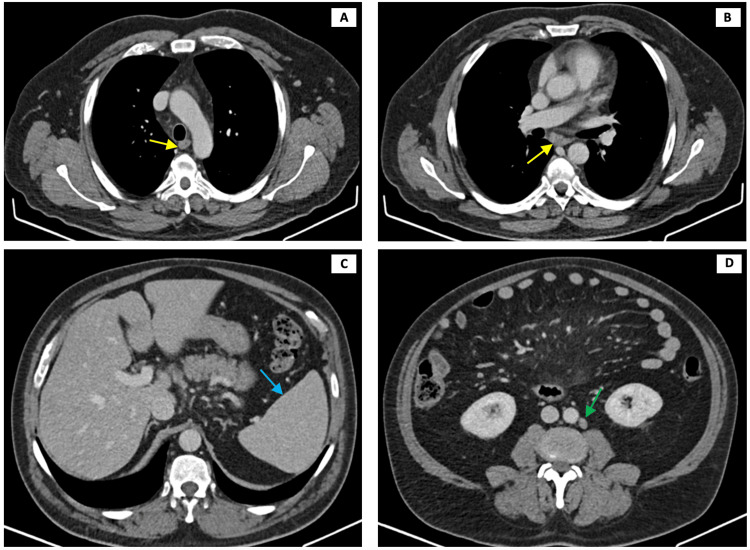
Thoracoabdominal CT scan showing mediastinal lymphadenopathy (A and B, yellow arrows), slight splenomegaly (C, blue arrow), and enlarged retroperitoneal lymph nodes (D, green arrow) CT: computed tomography

A thoracoabdominal computed tomography (CT) showed slight splenomegaly (140 mm in diameter), mediastinal and abdominal lymphadenopathy with many enlarged intra-abdominal and retroperitoneal lymph nodes, and a 6-mm diameter nonspecific pulmonary node in the right inferior lobe (Figure 1).

**Figure 2:**
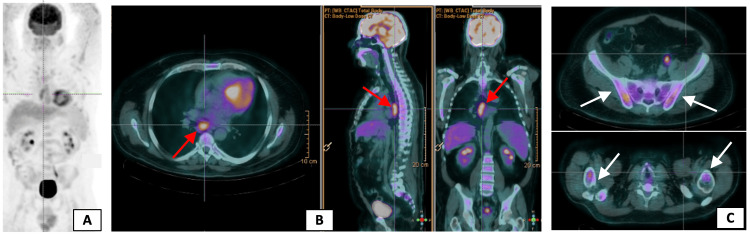
Whole-body FDG-18 PET-CT scan (A) showing a diffusely increased uptake in mediastinal lymphadenopathy (B, red arrows) and skeleton (C, white arrows) FDG-18: fludeoxyglucose-18; PET: positron-emission tomography; CT: computed tomography

The patient was admitted to the Internal Medicine ward for further investigation. The infectious study, which included human immunodeficiency virus, hepatitis B virus, hepatitis C virus, cytomegalovirus, Epstein-Barr virus, *Mycobacterium tuberculosis*, Syphilis, Toxoplasmosis, *Coxiella burnetii*, leptospirosis, leishmaniasis, borreliosis, and stool cultures, was negative. Additionally, the autoimmune workup, comprising antinuclear antibodies, antineutrophil cytoplasmic antibodies, rheumatoid factor, anticyclic citrullinated peptide, anti-double-stranded DNA antibodies, complement levels, and protein electrophoresis, yielded negative results. Tumor markers, including alpha-fetoprotein and prostate-specific antigen tests, also returned normal values, as did the angiotensin-converting enzyme level. Urine analysis shows no proteinuria or hematuria. A whole-body fludeoxyglucose-18 positron-emission tomography scan exhibited a diffusely increased uptake in the skeleton, mediastinal lymphadenopathy, and spleen (Figure 2).

**Figure 3:**
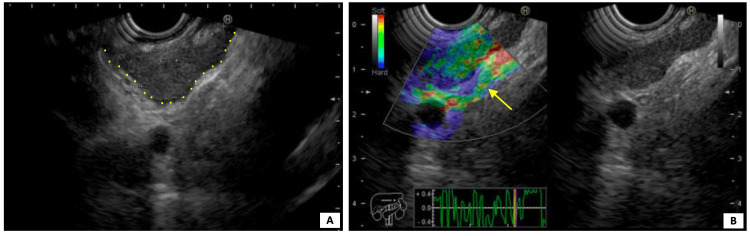
Endoscopic ultrasound-guided elastography of the larger mediastinal lymphadenopathy with 23 x 12 mm (A, yellow dots) showing a predominantly green pattern in the elastographic evaluation (B, yellow arrow), more in favor of a benign etiology

An endoscopic ultrasound was conducted to biopsy the larger mediastinal lymphadenopathy, measuring 23 x 12 mm (Figure 3), whose histopathological examination was compatible with a granulomatous inflammatory process.

**Figure 4:**
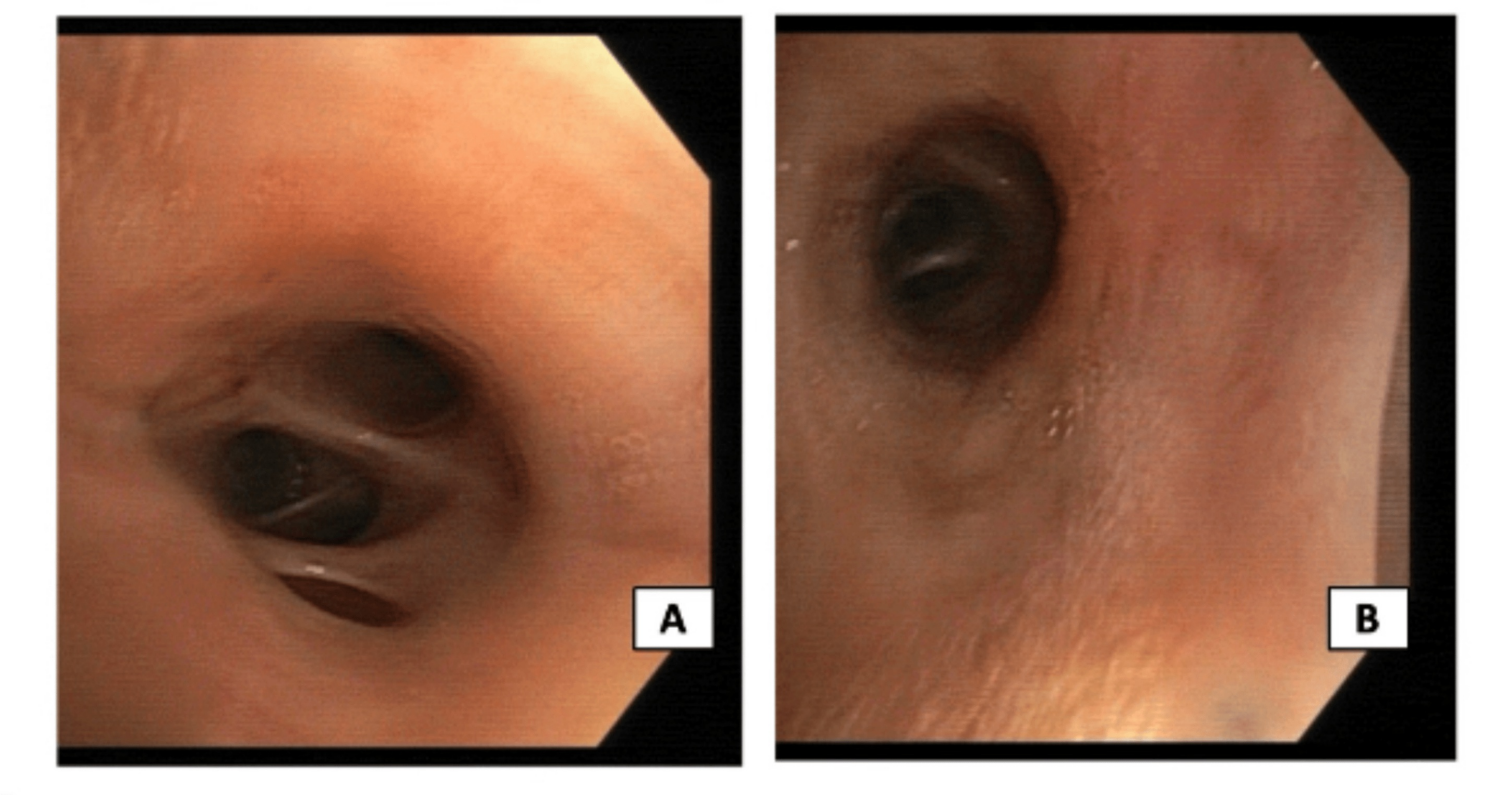
Normal bronchofibroscopy, showing no morphologic abnormalities. (A) Right upper lobe. (B) Left upper lobe

Bronchofibroscopy (Figure 4) with bronchoalveolar lavage and anatomopathological analysis of the pulmonary node also suggested an inflammatory process, excluding malignancy. Later, he underwent a bone marrow biopsy with a myelogram, immunophenotyping, and genetic analysis, excluding a myelomatous process. A recent colonoscopy performed before hospital admission showed a spastic and irritable colon, and upper endoscopy with gastric biopsy concluded chronic gastritis with a negative histology for *Helicobacter pylori*; no endoscopic studies were repeated during the hospitalization.

After an extensive investigation and presenting a twice-a-week fever pattern in response to paracetamol, the patient was discharged two months later and maintained follow-up in the Internal Medicine consult for additional investigation. He kept having self-limited episodes of fever (two to four per month) associated with myalgia, polyarthralgia, asthenia, and severe limitation in his daily living activities. An MEFV gene sequencing presented a heterozygous variant of uncertain clinical significance (c.330G>A). A genetic next-generation sequencing (NGS) panel for AIDs did not detect pathogenic variants that could explain his clinical presentation but identified a variant with potential clinical significance in the TREX1 gene, NM_033629.6:c.650G>A p.(Arg217Gln), exon 2, that should be interpreted within the clinical context. The patient had no family history of FMF or other AID.

Given the suspicion of FMF and after multidisciplinary discussion, the patient started colchicine, 1 mg three times daily. Regardless of the decreased inflammatory parameters (Table 1), he kept having recurrent episodes of fever even with the maximally tolerated dose of colchicine and association with other anti-inflammatory drugs, such as etoricoxib and paracetamol. No gastrointestinal symptoms were reported. Twelve months after starting colchicine, anakinra, 100 mg daily, was associated. Despite longer periods of apyrexia in the following three months (one to two per month) and lower inflammatory parameters (Table 1), he started having diarrhea and intense abdominal pain. After a multidisciplinary discussion, it was decided to suspend anakinra, continue treatment with colchicine, and ensure the resolution of gastrointestinal symptoms. The patient was evaluated by a medical geneticist, who repeated the NGS panel for AIDs, including exome analysis, which reported another variant of potential clinical significance in the CARD11 gene.

**Figure 5:**
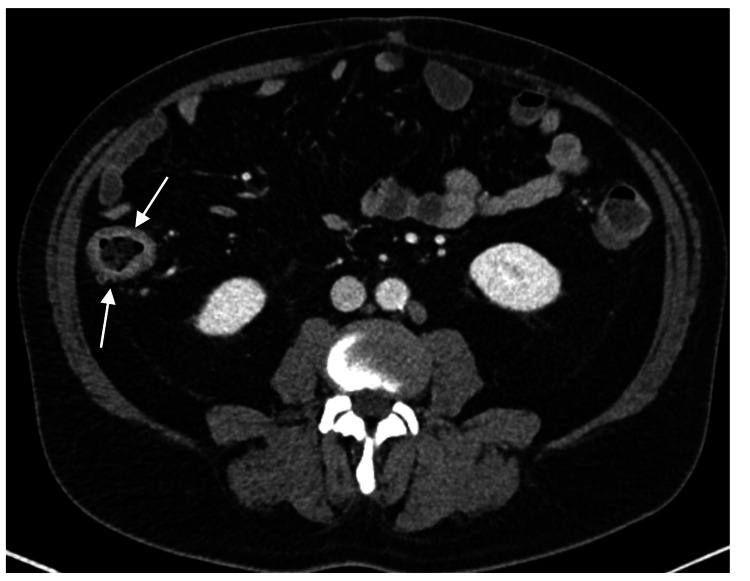
Abdominal CT scan, revealing ascending colon thickening walls (white arrows), suggestive of chronic colitis CT: computed tomography

Since the anakinra’s discontinuation and under colchicine, a gradually increasing frequency of fever episodes was noted (one to two per week) in the following six months, associated with intense diarrhea, asthenia, and anorexia within the last month. Evaluated in the emergency department, he presented with ascending inflammatory parameters, anemia, and high levels of fecal calprotectin (Table 1). An abdominal CT exhibited thickening walls of the transverse and ascending colon, possibly related to chronic colitis (Figure 5), and the patient was admitted to the Internal Medicine ward for additional investigation.

**Figure 6:**
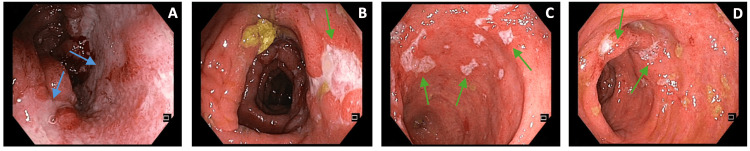
Colonoscopic findings. Deep ulcer (blue arrows) in the terminal ileum (A). Minor ulcers (green arrows) distributed throughout the colon: cecum (B and C) and rectum (D)

Infectious causes were again excluded. A colonoscopy showed a deep ileal ulcer (with 40 mm diameter) and other minor ulcers from de cecum up to 30 cm from the anal margin (with 5-15 mm diameter) (Figure 6): several biopsies were performed, whose histopathological examination confirmed polymorphic inflammatory infiltrates with cryptic lesions and formation of cryptic microabscesses.

**Figure 7:**
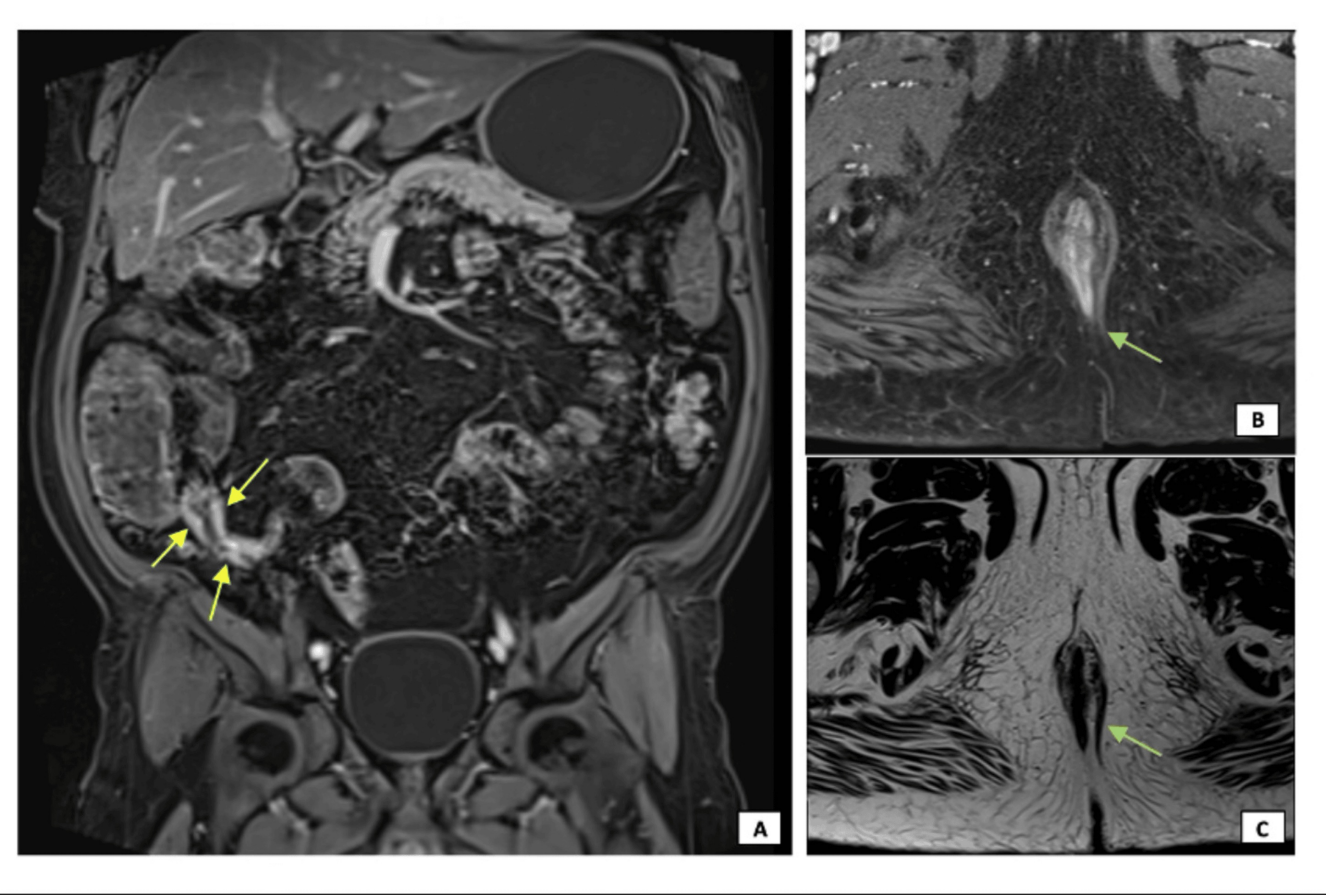
MR imaging of the pelvis and enterography demonstrating terminal ileum wall thickening, with contrast enhancement (A, yellow arrows), and an anal fistula with intersphincteric fistulous tract (B and C, green arrows), both findings suggestive of CD MR: magnetic resonance; CD: Crohn’s disease

Magnetic resonance (MR) imaging of the pelvis and MR enterography showed terminal ileum wall thickening with contrast enhancement and an anal fistula (Figure 7).

After presenting an inaugural diagnosis of ileocolic CD and discussing it with the Gastroenterology Department, the patient underwent a cycle of systemic corticosteroid therapy (intravenous methylprednisolone 62.5 mg per day for three days, then switched to prednisolone 40 mg per day with a withdrawal therapeutic scheme). This resulted in a good response, and the patient then began induction therapy with infliximab (5 mg/kg), a tumor necrosis factor-alpha inhibitor, which led to a significant improvement in gastrointestinal symptoms and effective control of fever. The medical geneticist requested a genetic evaluation of the patient’s progenitors, but the results are still pending.

## Discussion

FMF, being an AID, is caused by MEFV gene mutation and is characterized by self-limited episodes of fever and systemic inflammation, usually with pleural and abdominal serositis and arthritis [2]. Despite its clinical diagnosis being based on Tel-Hashomer criteria, it can be a challenging one, especially in patients with atypical symptoms, presenting with MEFV gene mutations of uncertain clinical significance and therapeutic failure with both colchicine and anakinra, as was the case of our patient. Most FMF-causing MEFV variants are founded on exons 2 and 10. Despite 85% of FMF patients having one of the three most frequent mutations (M680I, M949V, and V726A), there have been identified and reported more than 400 variants, most of them of unknown clinical significance, in the Infevers database (https://infevers.umai-montpellier.fr/web/search.php?n=1) [7]. Concerning FMF treatment, 20%-40% of patients display partial remission, and only 5%-10% do not respond to a maximally tolerated colchicine dose [2,5]. Although IL-1 inhibitors, such as anakinra, proved to have significantly higher efficacy in colchicine-resistant patients, cases reporting patients with no response to anakinra are rare [8,9].

FMF and IDB, specifically CD, are both inflammatory disorders characterized by fever, abdominal pain, arthralgia, obstipation, and diarrhea. Splenomegaly and abdominal lymphadenopathy are usually found in FMF patients [5], as seen in our patient.

Presumably, due to the dysregulation in the immune system, an association between FMF and CD has been demonstrated in a few studies. The protein encoded by the MEFV gene, pyrin, has been shown to interact with the gene product of NLRP3, NALP3/cryopyrin, an important active member of inflammasome that has been associated with CD susceptibility [3,10]. Recent studies also verified that CD seems to be more prevalent in patients with FMF, manifesting a later onset of gastrointestinal symptoms, with higher attack frequency and more severe complications [11,12], as supported by our case. Additionally, there are some cases reporting gastrointestinal involvement in FMF mimicking CD, mentioning patients presented with fever, gastrointestinal symptoms, and enterocolitis in imaging exams but without typical endoscopic or other clinical features of CD and whose diagnosis and treatment of FMF was delayed due to an alleged initial diagnosis of CD [6,13,14]. Very few cases describe coexisting FMF and CD, all of them being children or young adults [15]. Ghaffar and Elsayed presented a child with FMF, apparently colchicine-resistant and whose symptoms of CD were initially masked by the resistant fever of FMF, being later diagnosed with concomitant CD and only showing clinical improvement when started targeted therapy for CD [16]. Biological therapies, such as infliximab, have proved to be very effective in treating moderate-to-severe CD [17], as demonstrated in our patient.

## Conclusions

FMF, the most frequent AID, can be associated with other inflammatory disorders, such as IBD. While the gastrointestinal involvement in FMF is not the most typical presentation, both FMF and IBD can share clinical features that challenge their diagnosis and treatment.

This case highlights the complexity and diagnostic and therapeutic challenges faced when these two diseases coexist, particularly when atypical manifestations are the initial presentation. It also emphasizes the importance of a multidisciplinary approach, including genetic counseling, to improve diagnostic accuracy and obtain better clinical outcomes.
